# Supplementary material for: Real-time brain-machine interface in non-human primates achieves high-velocity prosthetic finger movements using a shallow feedforward neural network decoder
Source: Nat Commun. 2022 Nov 12;13:6899. doi: 10.1038/s41467-022-34452-w (PMC9653378; doi:10.1038/s41467-022-34452-w)
Supplement: Supplementary file 1 — Description of Additional Supplementary Files [file 41467_2022_34452_MOESM1_ESM.pdf]

File name: Supplementary Movie 1

Description: RK for a random finger task. The video captures the average performance of 992 the RK as measured by throughput (1.4 bps) over the two days of testing for Monkey N when 993 comparing RK and RN.

File name: Supplementary Movie 2

Description: RN for a random finger task. The video captures the average performance of 996 the RN decoder as measured by throughput (2.3 bps) over the two days of testing for Monkey N 997 when comparing RK and RN.

File name: Supplementary Movie 3

Description: RN for a center-out finger task. The video captures the peak performance of 1000 the RN decoder as measured by throughput (3.2 bps) in one day of testing of the RN for Monkey 1001 N on a typical center-out finger task.
