# Supplementary material for: Real-time brain-machine interface in non-human primates achieves high-velocity prosthetic finger movements using a shallow feedforward neural network decoder
Source: Nat Commun. 2022 Nov 12;13:6899. doi: 10.1038/s41467-022-34452-w (PMC9653378; doi:10.1038/s41467-022-34452-w)
Supplement: Supplementary file 5 — Reporting Summary [file 41467_2022_34452_MOESM5_ESM.pdf]

nature portfolio

Corresponding author(s): Cynthia A. Chestek

Last updated by author(s): Oct 10, 2022

Reporting Summary

Nature Portfolio wishes to improve the reproducibility of the work that we publish. This form provides structure for consistency and transparency in reporting. For further information on Nature Portfolio policies, see our Editorial Policies and the Editorial Policy Checklist.

Statistics

For all statistical analyses, confirm that the following items are present in the figure legend, table legend, main text, or Methods section.

n/a	Confirmed	
☐	☒	The exact sample size (*n*) for each experimental group/condition, given as a discrete number and unit of measurement
☐	☒	A statement on whether measurements were taken from distinct samples or whether the same sample was measured repeatedly
☐	☒	The statistical test(s) used AND whether they are one- or two-sided<br>*Only common tests should be described solely by name; describe more complex techniques in the Methods section.*
☐	☒	A description of all covariates tested
☒	☐	A description of any assumptions or corrections, such as tests of normality and adjustment for multiple comparisons
☐	☒	A full description of the statistical parameters including central tendency (e.g. means) or other basic estimates (e.g. regression coefficient) AND variation (e.g. standard deviation) or associated estimates of uncertainty (e.g. confidence intervals)
☐	☒	For null hypothesis testing, the test statistic (e.g. *F*, *t*, *r*) with confidence intervals, effect sizes, degrees of freedom and *P* value noted<br>*Give P values as exact values whenever suitable.*
☒	☐	For Bayesian analysis, information on the choice of priors and Markov chain Monte Carlo settings
☒	☐	For hierarchical and complex designs, identification of the appropriate level for tests and full reporting of outcomes
☒	☐	Estimates of effect sizes (e.g. Cohen's *d*, Pearson's *r*), indicating how they were calculated

Our web collection on statistics for biologists contains articles on many of the points above.

Software and code

Policy information about availability of computer code

Data collection	Neural data was collected using Utah arrays connected to the Cerebus neural signal processor (NSP, Blackrock Microsystems). Data were then transmitted to a PC running the xPC Target environment version 2012b (Mathworks, Natick, MA). Data were then sent to a connected computer with one 2070 super NVIDIA GPU (NVIDIA, Santa Clara, CA). This computer executed commands in Python (v3.7, https://www.python.org/) using the PyTorch library (v1.4, https://pytorch.org/). When training the network for online decoding, the neural network was optimized over 3500 iterations using the Adam optimization algorithm43 (torch.optim.Adam) with a learning rate of 10-4, weight decay of 10-2, and momentum of 0.9.
Data analysis	Data analysis was performed using computers with built-in code and customized code in Matlab (Mathworks, Natick, MA) versions R2017a, R2018a, PyCharm 2020-2022 and Python v3.7/v3.9, PyTorch v1.5/v1.12. Built-in functions utilized include:ttest.m, ttest2.m, anova1.m, numpy.correlate. Computer code will be available on lab website.

For manuscripts utilizing custom algorithms or software that are central to the research but not yet described in published literature, software must be made available to editors and reviewers. We strongly encourage code deposition in a community repository (e.g. GitHub). See the Nature Portfolio guidelines for submitting code & software for further information.

Data

Policy information about availability of data

All manuscripts must include a data availability statement. This statement should provide the following information, where applicable:

- Accession codes, unique identifiers, or web links for publicly available datasets
- A description of any restrictions on data availability
- For clinical datasets or third party data, please ensure that the statement adheres to our policy

The source data generated in this study have been provided within this paper, as supplementary data with this manuscript, and also available on the lab website [https://chestekresearch.engin.umich.edu/data-and-resources/]. The raw datasets used for this study are too large to be publicly shared, yet they are available for research purposes from the corresponding author on reasonable request.

Human research participants

Policy information about studies involving human research participants and Sex and Gender in Research.

Reporting on sex and gender	n/a
Population characteristics	n/a
Recruitment	n/a
Ethics oversight	n/a

Note that full information on the approval of the study protocol must also be provided in the manuscript.

Field-specific reporting

Please select the one below that is the best fit for your research. If you are not sure, read the appropriate sections before making your selection.

☒ Life sciences ☐ Behavioural & social sciences ☐ Ecological, evolutionary & environmental sciences

For a reference copy of the document with all sections, see nature.com/documents/nr-reporting-summary-flat.pdf

Life sciences study design

All studies must disclose on these points even when the disclosure is negative.

Sample size	The number of animals used was two, which is a standard number of typical non-human primate studies. Given the reproducibility of these results, no further animal experiments are deemed necessary. Validation of results was conducted over multiple days to acquire an abundance of data. No a priori power analyses were performed because multiple days in two animals provides more than enough power to validate claims, as is typical in non-human primate studies.
Data exclusions	None
Replication	Experimental findings were confirmed in two animals over multiple days.
Randomization	Randomization of decoders was not needed because decoders were used in both animals.
Blinding	Blinding with regards to animal experiments was not implemented as the experiments were automated. Regarding data analysis, the same computer code was used to analyze the results of all decoders. No further blinding during the analysis was implemented.

Reporting for specific materials, systems and methods

We require information from authors about some types of materials, experimental systems and methods used in many studies. Here, indicate whether each material, system or method listed is relevant to your study. If you are not sure if a list item applies to your research, read the appropriate section before selecting a response.

Materials & experimental systems

n/a	Involved in the study
☒	☐ Antibodies
☒	☐ Eukaryotic cell lines
☒	☐ Palaeontology and archaeology
☐	☒ Animals and other organisms
☒	☐ Clinical data
☒	☐ Dual use research of concern

Methods

n/a	Involved in the study
☒	☐ ChIP-seq
☒	☐ Flow cytometry
☒	☐ MRI-based neuroimaging

Animals and other research organisms

Policy information about studies involving animals; ARRIVE guidelines recommended for reporting animal research, and Sex and Gender in Research

Laboratory animals	The study used two adult male rhesus macaques, both were 9 years old at the time of the study.
Wild animals	The study did not involve wild animals.
Reporting on sex	The study did not involve female animals.
Field-collected samples	The study did not involve samples collected from the field.
Ethics oversight	The study as approved by the University of Michigan Institutional Animal Care and Use Committee, PRO00010076.

Note that full information on the approval of the study protocol must also be provided in the manuscript.
